# A universal genome sequencing method for rotavirus A from human fecal samples which identifies segment reassortment and multi-genotype mixed infection

**DOI:** 10.1186/s12864-017-3714-6

**Published:** 2017-04-24

**Authors:** Tran Thi Ngoc Dung, Pham Thanh Duy, October M. Sessions, Uma K. Sangumathi, Voong Vinh Phat, Pham Thi Thanh Tam, Nguyen Thi Nguyen To, Tran My Phuc, Tran Thi Hong Chau, Nguyen Ngoc Minh Chau, Ngoc Nguyen Minh, Guy E. Thwaites, Maia A. Rabaa, Stephen Baker

**Affiliations:** 1 The Hospital for Tropical Diseases, Wellcome Trust Major Overseas Programme, Oxford University, Clinical Research Unit, 764 Vo Van Kiet, Quan 5, Ho Chi Minh City, Vietnam; 2 Duke – National University of Singapore, Singapore, Singapore; 3 Children’s Hospital 2 (CH2), Ho Chi Minh City, Vietnam; 4 Centre for Tropical Medicine, Oxford University, Oxford, UK; 5 The London School of Hygiene and Tropical Medicine, London, UK

**Keywords:** Rotavirus A, Genomics, Phylogenetics, Genome sequencing, Reassortment, Co-infection, Antibody capture

## Abstract

**Background:**

Genomic characterization of rotavirus (RoV) has not been adopted at large-scale due to the complexity of obtaining sequences for all 11 segments, particularly when feces are used as starting material.

**Methods:**

To overcome these limitations, we developed a novel RoV capture and genome sequencing method combining commercial enzyme immunoassay plates and a set of routinely used reagents.

**Results:**

Our approach had a 100% success rate, producing >90% genome coverage for diverse RoV present in fecal samples (Ct < 30).

**Conclusions:**

This method provides a novel, reproducible and comparatively simple approach for genomic RoV characterization and could be scaled-up for use in global RoV surveillance systems.

**Trial registration (prospectively registered):**

Current Controlled Trials ISRCTN88101063. Date of registration: 14/06/2012

**Electronic supplementary material:**

The online version of this article (doi:10.1186/s12864-017-3714-6) contains supplementary material, which is available to authorized users.

## Background

The control of diarrheal diseases remains a constant public health challenge; current estimates predict that diarrhea results in approximately 800,000 deaths and 90,000 disability adjusted life years (DALYs) globally per year [1, 2]. The greatest burden of disease, and consequently the biggest impact on DALYs, arises in children under the five years of age residing in countries with a low economic index [3]. Diarrhea is a complex syndrome that can be induced by a number of perturbations of the gastrointestinal tract, but the disease is generally associated with specific viruses, bacteria and parasites that induce diarrhea via differing mechanisms. Despite the availability of efficacious vaccines against rotavirus A (RoV), this ubiquitous, highly virulent and extremely transmissible virus remains the most common cause of diarrhea in children under the age of two years globally [4, 5]. RoV is the suspected etiological agent in 39% and 45% of all hospital admissions related to diarrhea globally and in Asia, respectively. In our setting in Vietnam, where vaccine uptake has been limited, RoV is estimated to be responsible for between 40 and 60% of all childhood diarrheal infections requiring hospitalization [6, 7].

RoV is a non-enveloped virus and a member of the Reoviridae with a genome comprised of 11 segments (g1-g11) of double-stranded RNA (dsRNA) of differing lengths [8]. These 11 segments encode the six structural (NSP1-NSP6) and six non-structural proteins (VP1-4, VP6 and VP7) that constitute a functional, infectious virion. Currently, sequence variation within two of the genes encoding the outer viral capsid proteins (VP7 and VP4) permits a basic differentiation of RoV strains [9]. The sequences of the VP7 (glycoprotein) and the VP4 (protease-sensitive protein) genes define the G-type and P-type, respectively. G1P[8] is consistently the most frequent RoV A G/P type isolated from symptomatic humans globally, but 27 other G types and 37 alternative P types have been described [10, 11].

G/P typing has historically been considered to be adequate for RoV surveillance, epidemiology and for the identification of escapees during vaccine efficacy studies [12–14]. However, sequencing of just 2/11 genome segments disregards approximately 81% (15,138 bp/18,550 bp considering the SA11 reference sequence [15]) of the genome that exists outside these capsid genes, thus limiting our phylogenetic understanding of the other genome segments and the evolutionary processes that may be active across the RoV genome. To address this limitation, a more complex RoV classification system using all eleven genomic segments has been established, resulting in a whole genome nomenclature of Gx-P[x]-Ix-Rx-Cx-Mx-Ax-Nx-Tx-Ex-Hx, representing VP7-VP4-VP6-VP1-VP2-VP3-NSP1-NSP2-NSP3-NSP4-NSP5, respectively [10].

Currently, the purification of pure RoV RNA suitable for direct genome sequencing is largely dependent on viral culture. This method is laborious, unreliable and induces bias for sequencing of high yield, cultivable viral particles. For RoV genotyping, RoV RNA is extracted directly from fecal samples and PCR amplification is performed on the VP4 and VP7 regions. This method is fundamentally unsuitable for genome sequencing via next generation sequencing owing to the high amount of contaminating (non-viral) nucleic acid in the sample, and produces low yields of RoV-specific sequence (in comparison to other species) upon next generation sequencing. Despite the introduction of a whole genome nomenclature for RoV, whole genome sequencing is yet to be universally adopted for routine identification, surveillance and phylogenetic studies. This is because the whole genome sequencing of RoV is far from straightforward and relies on the independent amplification of each segment from a positive fecal sample. The combination of dsRNA, fecal extraction as an amplification template, untypeable viruses and highly polymorphic sequences add to this complexity. The presence of mixed infection of a single sample with multiple RoV, reported in approximately 10% of infections in developed countries [16, 17] and 21–48% in low income countries [18, 19], further complicates RoV genotyping and sequencing efforts. The use of Sanger sequencing methods, which generate a single consensus sequence based on nucleotide abundance, does not permit the detection of genetic variation or the presence of multiple viruses within a sample, both of which may bias inference related to RoV infection and evolution. Random amplification combined with next generation sequencing (NGS) methods can be utilized overcome many of the complications that prevent the generation of adequately characterised RoV gene sequences representative of the complete diversity present in a single sample. However, there are still barriers; feces is a complex starting material, containing inhibitors and a wide diversity of organic material, and it is likely that RoV dsRNA will be of low yield in comparison to nucleic acid from other more copious viruses, prokaryotes, and eukaryotic cells found in the gastrointestinal tract. To overcome these issues, we present here a novel method suitable for the generation of whole genome sequences of RoV directly from human fecal specimens.

## Results

### A novel method for the purification of rotavirus nucleic acid from fecal samples

**Fig. 1:**
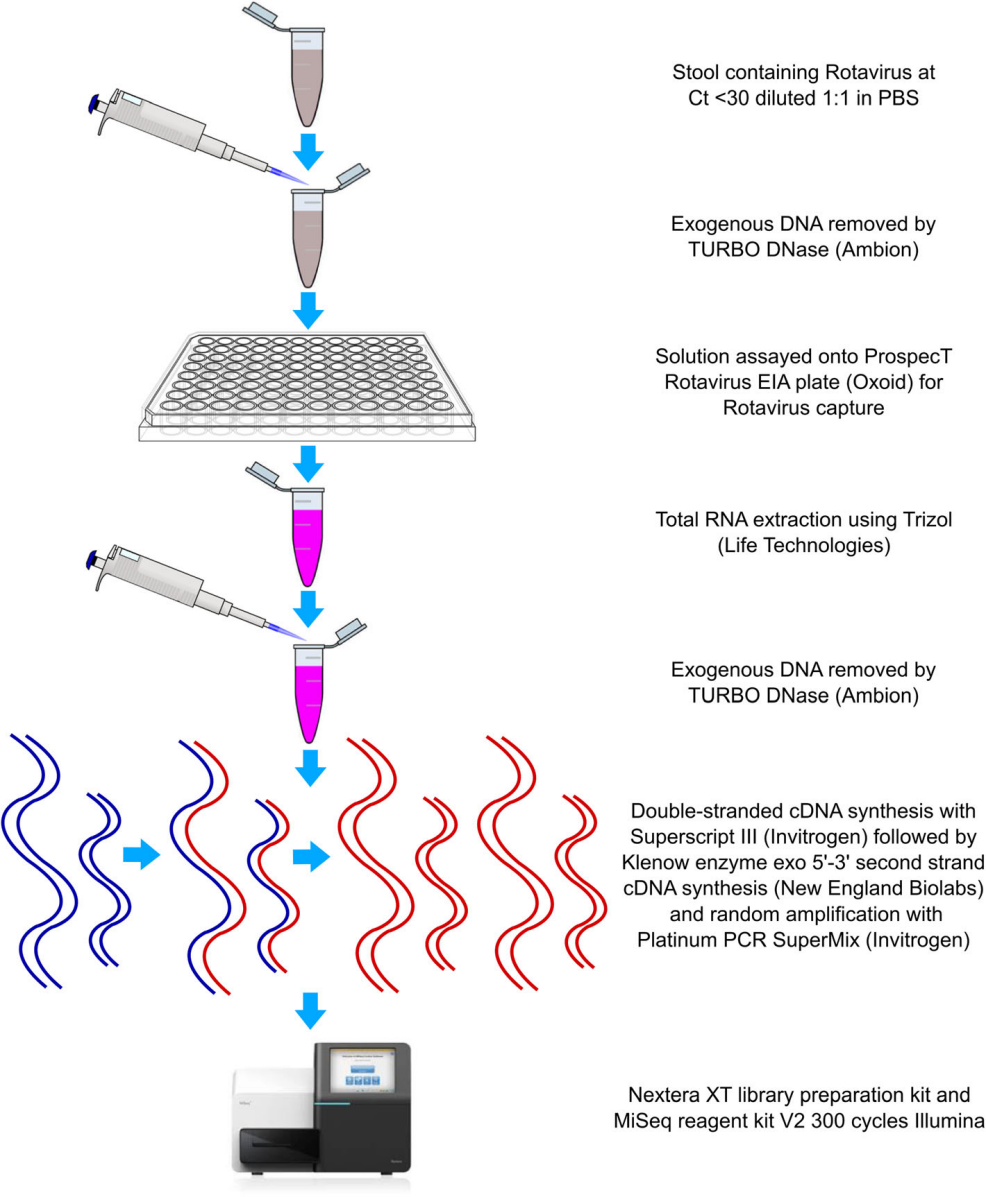
The procedure for rotavirus capture and nucleic acid preparation prior to genome sequencing. Flow diagram describing the major protocol steps required for the purification of RoV and the amplification of RoV specific nucleic acid for next generation genome sequencing. Image of Illumina MiSeq obtained from https://assets.illumina.com/content/dam/illumina-marketing/images/systems/miseqdx/web-graphic-miseq-front-comparison-chart.jpg. Clipart image of pipette obtained from http://www.clker.com/clipart-pipette-with-tip.html. Clipart image of 96-well plate obtained from http://cyberuse.com/96-well-plate-template.html. Clipart image of Eppendorf tube obtained from http://www.clker.com/clipart-eppendorf-tube-with-open-cap-1.html and edited in Adobe Illustrator

To address the limitations surrounding RoV nucleic acid purification, we developed a new method utilizing commercial enzyme immunoassay (EIA) plates to capture RoV particles suitable for RNA extraction and amplification. This procedure is described in detail in the Methods; Fig. 1 shows a flow diagram outlining the various laboratory steps. Briefly, fecal samples testing positive for RoV by Reverse Transcriptase (RT)-PCR were captured using EIA plates, exogenous nucleic acids removed, and RNA extracted from the captured viral particles. In order to prepare the purified RoV RNA for genome sequencing the RNA was converted to double-stranded cDNA prior to standard library preparation necessary for Illumina sequencing.

### Rotavirus purification for genome sequencing is dependent on viral load

We first performed and validated the RoV purification method on a human fecal sample that contained a high yield (Ct value = 15, estimated 1 × 10^8^ copies) of the most common RoV genotype in human infections, G1P[8] (Table 1). We found that purification of viral particles on EIA plates and the conversion to cDNA substantially reduced the yield of RoV RNA, producing sequential Ct values of 23 and 28.5 at each step, respectively (Table 1). However, after random amplification [20], the Ct value was restored to 15 and subsequent genome sequencing on an Illumina MiSeq machine produced >600,000 reads, of which >50% could be attributed to RoV (i.e. <50% contamination with non-RoV sequences) with >90% genome coverage (Table 1). Predictably, after performing several serial dilutions, we found the RoV purification method was highly sensitive to viral load, with minimal RoV sequences produced at a starting Ct value of 25 and no RoV sequences produced when the initial starting Ct value was >30 (estimated <1 × 10^3^ copies) (Table 1).

**Table 1:** Concentration of rotavirus required for genome sequence generation

Sample	Initial input		Post-viral capture		Double-stranded cDNA		Random amplification		Genome sequencing			
	Ct value	Target copies	Ct value	Target copies	Ct value	Target copies	Ct value	Target copies	Total reads	Number of RoV reads	Proportion of RoV reads (%)	RoV genome coverage (%)
Neat	15	1 × 10^8^	23	6 × 10^5^	28.53	1 × 10^4^	14.97	1 × 10^8^	6 × 10^5^	3 × 10^5^	54.5	90.4
Dilution (2^−5^)	20	4 × 10^6^	28	2 × 10^4^	34.9	2 × 10^2^	19.59	6 × 10^6^	7 × 10^5^	2 × 10^5^	20.7	81.3
Dilution (2^−10^)	25	1 × 10^5^	33	6 × 10^2^	37.44	3 × 10^1^	23.9	3 × 10^5^	8 × 10^5^	5 × 10^3^	0.7	49.3
Dilution (2^−15^)	30	7 × 10^3^	36	8 × 10^1^	Negative	0	Negative	0	8 × 10^5^	6 × 10^2^	0.07	26.7
Dilution (2^−16^)	32	1 × 10^3^	Negative	0	Negative	0	Negative	0	0	0	0	0

### Validation of method for the genome sequencing of differing rotavirus genotypes

We next aimed to assess the performance of the RoV purification and sequencing method on a range (*n* = 26) of samples containing common (G1P[8]) and less common RoV genotypes, these included more “exotic” genotypes such as G26P[19] and previously untypeable RoV positive samples (samples for which a G/P type could not be determined using the conventional PCR amplification method). Only samples with a primary Ct value for RoV of <25 were chosen. Random amplification and Illumina sequencing of all 26 samples, including those containing untypable RoV, yielded large amounts of sequences associated with RoV. The number of RoV reads ranged from ~66,000 to >1,500,000 (median = 761,135) and the percentage of reads corresponding with RoV in the final sequencing data ranged from 7.4% to >97% (Table 2).

**Table 2:** Concentration of rotavirus in samples at each step of the capture and amplification process

Sample ID	GP type	Initial input		Post-viral capture		Double-stranded cDNA		Random amplification		Genome sequencing	
		Ct value	Target copies	Ct value	Target copies	Ct value	Target copies	Ct value	Target copies	Total reads	RoV reads (% output)
VN-0002	G1P[8]	13.25	4 × 10^8^	22.75	7 × 10^5^	28.25	2 × 10^4^	16.3	5 × 10^7^	1,009,250	951,969 (94.3)
VN-0006	G1P[8]	15.28	1 × 10^8^	24.78	2 × 10^5^	30.28	4 × 10^3^	18.02	2 × 10^7^	1,102,774	932,674 (84.6)
VN-0013	G1P[8]	14.07	2 × 10^8^	23.57	4 × 10^5^	29.07	9 × 10^3^	23.18	5 × 10^5^	1,172,366	1,087,933 (93.0)
VN-0057	G1P[8]	10.31	3 × 10^9^	19.81	5 × 10^6^	25.31	1 × 10^5^	16.92	4 × 10^7^	1,269,282	910,567 (71.7)
VN-0030	G1P[8]	11.66	1 × 10^9^	21.16	2 × 10^6^	26.66	5 × 10^4^	18.45	1 × 10^7^	1,064,026	78,217 (7.4)
VN-0062	G1P[8]	8.3	1 × 10^10^	20.45	3 × 10^5^	35.65	1 × 10^2^	20.55	3 × 10^6^	1,037,480	778,628 (75.1)
VN-0324	G1P[8]	17.54	2 × 10^7^	27.04	4 × 10^4^	32.54	9 × 10^2^	20.12	4 × 10^6^	1,279,046	1,042,325 (81.5)
VN-0365	G1P[8]	13.82	3 × 10^8^	23.32	5 × 10^5^	28.82	1 × 10^4^	15.89	7 × 10^7^	1,305,760	1,271,769 (97.4)
VN-0299	G1P[8]	14.22	2 × 10^8^	23.72	4 × 10^5^	29.22	8 × 10^3^	27.8	2 × 10^4^	1,266,286	1,149,123 (90.8)
VN-0172	G1P[8]	14.3	1 × 10^10^	23.80	3 × 10^5^	29.3	8 × 10^3^	19.63	6 × 10^6^	1,816,574	1,298,619 (71.5)
VN-0341	G1P[8]	12.14	9 × 10^8^	21.64	1 × 10^6^	27.14	3 × 10^4^	20.61	3 × 10^6^	1,668,438	1,514,101 (90.8)
VN-0221	UT	18.38	1 × 10^7^	27.88	2 × 10^4^	33.38	5 × 10^2^	30.63	3 × 10^3^	1,479,948	336,552 (22.7)
VN-0186	UT	17.7	2 × 10^7^	27.20	3 × 10^4^	32.7	8 × 10^2^	24.78	2 × 10^5^	1,459,470	1,440,853 (98.7)
VN-0175	UT	24.25	2 × 10^5^	33.75	4 × 10^2^	39.25	9 × 10^1^	29.96	5 × 10^3^	1,306,208	1,138,961 (87.2)
VN-0181	UT	25	1 × 10^5^	34.50	2 × 10^2^	40	5 × 10^1^	30.44	4 × 10^3^	1,285,912	140,328 (10.9)
VN-0222	G2P[4]	18.76	1 × 10^7^	28.26	2 × 10^4^	33.76	4 × 10^2^	23	6 × 10^5^	1,289,724	1,204,896 (93.4)
VN-0321	G2P[4]	21.62	1 × 10^6^	31.12	2 × 10^3^	36.62	5 × 10^1^	26	7 × 10^4^	1,113,180	724,701 (65.1)
VN-0326	G2P[4]	13.5	4 × 10^8^	23.00	6 × 10^5^	28.5	1 × 10^4^	27.48	3 × 10^4^	426,240	347,019 (81.4)
VN-0058	G26P[19]	17	1 × 10^10^	26.50	5 × 10^4^	32	1 × 10^3^	28.98	1 × 10^4^	1,434,422	739,222 (51.5)
VN-0132	G3P[4]	16.66	4 × 10^7^	26.16	7 × 10^4^	31.66	2 × 10^3^	23.68	4 × 10^5^	1,316,574	743,642 (56.5)
VN-1221	G2P[4]	13.25	4 × 10^8^	22.75	7 × 10^5^	28.25	1 × 10^3^	16.26	1 × 10^4^	700,632	555,195 (79.2)
VN-0074	G1P[8]	14.8	2 × 10^8^	24.30	2 × 10^5^	29.8	6 × 10^3^	16.87	4 × 10^7^	991,014	153,654 (15.5)
VN-0140	G1P[8]	13.21	4 × 10^8^	22.71	7 × 10^5^	28.21	1 × 10^3^	15.7	1 × 10^4^	665,478	578,601 (87.0)
VN-0196	G9P[4]	16.18	6 × 10^7^	25.68	9 × 10^4^	31.18	2 × 10^3^	17.28	3 × 10^7^	743,184	66,378 (8.9)
VN-3096	G1P[4]	13.52	4 × 10^8^	23.02	6 × 10^5^	28.52	1 × 10^3^	15.78	1 × 10^4^	567,266	381,270 (67.2)
VN-1604	G2P[4]	15.31	1 × 10^8^	24.81	2 × 10^5^	30.31	4 × 10^3^	17.55	2 × 10^7^	670,404	330,173 (49.3)

### Optimisation of rotavirus cDNA amplification for genome sequencing

**Fig. 2:**
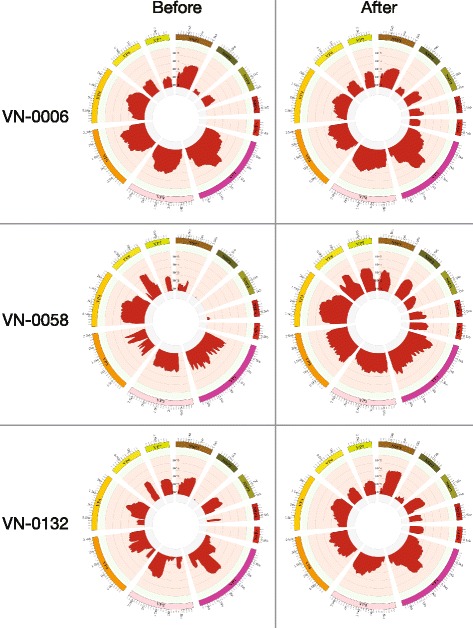
Rotavirus nucleic acid enrichment for whole genome sequencing. Circular plots showing the coverage (i.e. the amount of sequence produced across each segment) of each of the 11 RoV segments (highlighted) and the coverage depth of each of the segments after Illumina sequencing for three RoV-positive samples (top, middle, bottom). Plots show coverage and depth following viral capture and random amplification (left, before) and following viral capture and amplification with both random and specific primers (right, after)

We measured the ability of the method to produce sequences that covered all 11 segments of the RoV genome. We found that, even when the proportion of reads associated with RoV was low for some samples, that the genome coverage (per base) was high, with 20/26 of the RoV positive samples producing sequences corresponding with >90% coverage of reference genomes (median coverage, 92.5%; interquartile range (IQR), 90.7–94.7%). However, we additionally found that the resulting sequences were highly influenced by segment length. The VP1 (3,302 bp) and VP2 (2,690 bp) regions exhibited a median coverage of >99.9%, whilst the smaller segments, such as NSP4 (751 bp) and NSP5 (667 bp), exhibited a median coverage of only 77.4% (IQR, 61.5–92.3%) and 52.6% (IQR, 27.8–86.4%), respectively (Table 3). These data suggest a limitation of the random amplification procedure due to an amplification bias against the shorter RoV genome segments. The underrepresentation of the NSP genes (NSP2-5) and VP7 in the final genome sequences for three selected samples (VN-0006 (G1P[8]), VN-0058 (G26P[19]) and VN-0132 G3P[4])) can be observed in the sequence coverage plots in Fig. 2.

**Table 3:** Sequence coverage of rotavirus genome segments using viral capture and random amplification

Sample ID	GP type	Genome Coverage (%)	VP7	VP4	VP6	VP1	VP2	VP3	NSP1	NSP2	NSP3	NSP4	NSP5
			Segment length and coverage (%)										
VN-0002	G1P[8]	98.49	1062 (100)	2362 (100)	1356 (100)	3302 (100)	2690 (100)	2591 (100)	1497 (92.9)	1041 (98.3)	1104 (100)	701 (93.3)	567 (85.0)
VN-0006^a^	G1P[8]	91.95	1012 (95.3)	2338 (99.0)	988 (72.9)	3302 (100)	2488 (92.5)	2548 (98.3)	1597 (99.1)	919 (86.8)	1104 (100)	465 (61.9)	300 (45.0)
VN-0013	G1P[8]	99.61	1062 (100)	2359 (99.9)	1332 (98.2)	3302 (100)	2690 (100)	2591 (100)	1579 (98.0)	980 (92.5)	1101 (99.7)	714 (95.1)	606 (90.9)
VN-0057	G1P[8]	97.54	978 (92.1)	2328 (98.6)	1294 (95.4)	3302 (100)	2690 (100)	2575 (99.4)	1514 (94.0)	982 (92.7)	1121 (101.5)	626 (83.4)	606 (90.9)
VN-0030	G1P[8]	95.98	912 (85.9)	2312 (97.9)	1340 (98.8)	3302 (100)	2592 (96.4)	2585 (99.8)	1519 (94.3)	814 (76.9)	1104 (100)	749 (99.7)	579 (86.8)
VN-0062	G1P[8]	91.79	919 (86.5)	2345 (99.3)	913 (67.3)	3205 (97.1)	2690 (100)	2555 (98.6)	1500 (93.1	835 (78.8)	1104 (100)	589 (78.4)	375 (56.2
VN-0324	G1P[8]	94.57	1062 (100)	2359 (99.9)	1356 (100)	3302 (100)	2686 (99.9)	2591 (100)	1328 (82.4)	563 (53.2	1049 (95.0)	714 (95.1)	537 (80.5)
VN-0365	G1P[8]	93.87	961 (90.5)	2359 (99.9)	1204 (88.8)	3302 (100)	2690 (100)	2591 (100)	1539 (95.5)	976 (92.2	1041 (94.3)	530 (70.1)	184 (27.6)
VN-0299	G1P[8]	94.44	948 (89.3)	2314 (98.0)	1181 (87.1)	3215 (97.4)	2690 (100)	2591 (100)	1393 (86.5)	962 (90.8)	1008 (91.3)	440 (58.6)	0 (0)
VN-0172^a^	G1P[8]	88.00	956 (90.0)	2141 (90.6)	1043 (76.9)	3233 (97.9)	2629 (97.7)	2535 (97.8)	1416 (87.9)	570 (53.8)	1041 (94.3)	252 (33.6)	340 (51.0)
VN-0341^a^	G1P[8]	82.54	1012 (95.3)	2338 (99.9)	988 (72.9)	3302 (100)	2488 (92.5)	2548 (98.3)	1009 (62.6)	741 (70.0)	943 (85.4)	72 (9.6)	0 (0)
VN-0221^a^	UT	79.00	867 (81.6)	1945 (82.3)	950 (70.1)	3154 (95.5)	2137 (79.4)	2308 (89.1)	1483 (92.1	539 (50.9)	700 (63.4)	461 (61.4)	0 (0)
VN-0186	UT	99.07	1062 (100)	2359 (99.9)	1356 (100)	3302 (100)	2690 (100)	2591 (100)	1556 (96.6)	1059 (100)	1041 (94.3)	699 (93.1)	667 (100)
VN-0175	UT	91.01	950 (89.5)	2362 (100)	1123 (82.8)	3302 (100)	2690 (100)	2591 (100)	1350 (83.8)	717 (67.7)	1049 (95.0)	675 (89.9)	64 (9.6)
VN-0181^a^	UT	81.64	780 (73.4)	1678 (71.0)	1170 (86.3)	3123 (94.6)	2142 (79.6)	2436 (94.0)	1418 (88.0)	827 (78.1	935 (84.7)	432 (57.5)	667 (100)
VN-0222	G2P[4]	94.80	1031 (97.1)	2362 (100)	1324 (97.6)	3302 (100)	2690 (100)	2591 (100)	1455 (90.3)	898 (84.8)	1036 (93.8)	655 (87.2	210 (31.5)
VN-0321	G2P[4]	91.40	1044 (98.3)	2330 (98.6)	1144 (84.4)	3302 (100)	2637 (98.0)	2591 (100)	1466 (91.0)	860 (81.2	1058 (95.8)	350 (46.6)	176 26.4)
VN-0326	G2P[4]	90.63	952 (89.6)	2335 (98.9)	1083 (79.9)	3302 (100)	2690 (100)	2591 (100)	1263 (78.4)	649 (61.3)	960 (87.0)	589 (78.4)	362 (54.3)
VN-0058^a^	G26P[19]	82.00	800 (75.3)	2359 (99.9)	853 (62.9)	3302 (100)	2400 (89.2)	2490 (96.1)	304 (18.9)	890 (84.0)	709 (64.2)	641 (85.4)	331 (49.6)
VN-0132^a^	G3P[4]	81.00	953 (89.7)	1704 (72.1)	666 (49.1)	3300 (99.9)	2731 (101.5)	2504 (96.6)	1312 (81.4)	478 (45.1	1026 (92.9)	307 (40.9)	0 (0)
VN-1221	G2P[4]	93.83	1020 (96.0)	2334 (98.8)	1356 (100)	3290 (99.6)	2596 (96.5)	2471 (95.4)	1374 (85.3)	1040 (98.2	988 (89.5)	751 (100)	191 (28.6)
VN-0074	G1P[8]	92.09	1007 (94.8)	2062 (87.3)	1356 (100)	3067 (92.9)	2735 (101.7)	2093 (80.8)	1566 (97.2	1058 (99.9)	1074 (97.3)	574 (76.4)	495 (74.2)
VN-0140	G1P[8]	94.78	990 (93.2)	2193 (92.8)	1356 (100)	3302 (100)	2649 (98.5)	2452 (94.6)	1375 (85.4)	1059 (100)	985 (89.2	565 (75.2	660 (99.0)
VN-0196	G9P[4]	91.12	997 (93.9)	2281 (96.6)	1356 (100)	3237 (98.0)	2707 (100)	1925 (74.3)	1502 (93.2	966 (91.2	1012 (91.7)	481 (64.0)	444 (66.6)
VN-3096	G1P[4]	95.51	1029 (96.9)	2334 (98.8)	1356 (100)	3302 (100)	2641 (98.2)	2505 (96.7)	1390 (86.3)	1059 (100)	966 (87.5)	478 (63.6)	662 (99.3)
VN-1604	G2P[4]	92.49	1034 (97.4)	2334 (98.8)	1356 (100)	3252 (98.5)	2619 (97.4)	2347 (90.6)	1150 (71.4)	1056 (99.7)	1005 (91.0)	749 (99.7)	260 (38.9)

^a^Samples with low coverage chosen for resequencing using both random and specific primers (results shown in Table 5)

To enhance our ability to amplify entire RoV genomes from fecal samples, improve the coverage of the shorter genome segments and reduce contaminating sequences from other species, we aimed to enrich the amplification process prior to genome sequencing. We used a combination of the random primer FR26RVN and new FR26RV specific primers, exploiting conserved terminal sequences for each segment to generate specific primers for each of 11 segments (Table 4). We selected seven samples (VN-0172, VN-0341, VN-0221, VN-0181, VN-0058, VN-0132 and VN-0006) that generated incomplete sequences for the shorter genome segments using the random amplification primers alone and repeated the amplification step with the new primer combinations. The addition of the primer cocktail substantially improved the production of complete genome sequences. Specifically, we were able to generate enhanced coverage of all of the short segments, with the median coverage of NSP2, NSP4 and NSP5 increasing from 70 to 99.9%, from 57.5 to 86.8% and 45 to 86%, respectively (Tables 3 and 5). The coverage plots in Fig. 2 show the effect of the modified primer cocktail on whole genome amplification and segment sequencing for three selected samples: VN-0006, VN-0058 and VN-0132.

**Table 4:** Primers used for rotavirus genome enrichment amplification

Primer name	Primer sequences (5'-3')
FR20RV Modified FR26RVN FR26RV-VP7-F FR26RV-VP7-R	GCCGGAGCTCTGCAGATATC GCCGGAGCTCTGCAGATAT-non ribosomal hexanucleotides [2] GCCGGAGCTCTGCAGATATCGGCTTTAAAAG GCCGGAGCTCTGCAGATATCGGTCACATCATA
FR26RV-VP4-F FR26RV-VP4-R FR26RV-VP4-F2 FR26RV-VP4-R2	GCCGGAGCTCTGCAGATATCGGCTATAAAATG GCCGGAGCTCTGCAGATATCGGTCACATCCTC GCCGGAGCTCTGCAGATATCTGGCTTCGCCAT GCCGGAGCTCTGCAGATATCATTTCGGACCAT
FR26RV-VP6-F FR26RV-VP6-R	GCCGGAGCTCTGCAGATATCGGCTTTWAA ACG GCCGGAGCTCTGCAGATATCGGTCAC ATCCTC
FR26RV-VP1-Fb FR26RV-VP1-Rb	GCCGGAGCTCTGCAGATATCGGCTATTAAAGC GCCGGAGCTCTGCAGATATCGGTCACATCT
FR26RV-VP2-Fc FR26RV-VP2-Rc	GCCGGAGCTCTGCAGATATCGGCTATTAAAGG GCCGGAGCTCTGCAGATATCGTCATATCTCCA
FR26RV-VP3-Fe FR26RV-VP3-Re	GCCGGAGCTCTGCAGATATCGGCTWTTAAAGC GCCGGAGCTCTGCAGATATCGGTCACATCATG
FR26RV-NSP1-F FR26RV-NSP1-R	GCCGGAGCTCTGCAGATATCGGCTTTTTTTTATG GCCGGAGCTCTGCAGATATCGGTCACATTTTATGC
FR26RV-NSP2-F FR26RV-NSP2-R	GCCGGAGCTCTGCAGATATCGGCTTTTAAAGC GCCGGAGCTCTGCAGATATCGGTCACATAAGC
FR26RV-NSP3-F FR26RV-NSP3-R FR26RV-NSP3-R2	GCCGGAGCTCTGCAGATATCGGCTTTTAATGC GCCGGAGCTCTGCAGATATCACATAACGCCCCTAT GCCGGAGCTCTGCAGATATCGGTCACATAACG
FR26RV-NSP4-F1 FR26RV-NSP4-F2 FR26RV-NSP4-R1 FR26RV-NSP4-R2	GCCGGAGCTCTGCAGATATCGGCTTTTAAAAGTTC GCCGGAGCTCTGCAGATATCGCDTCAATTCCAAC GCCGGAGCTCTGCAGATATCTAAGACCRTTCC GCCGGAGCTCTGCAGATATCCTCCGCCGCTTCCGA
FR26RV-NSP5-F1 FR26RV-NSP5-F2 FR26RV-NSP5-R1 FR26RV-NSP5-R2	GCCGGAGCTCTGCAGATATCGGCTTTTAAAGCGCT GCCGGAGCTCTGCAGATATCGGCTTTTAAAGCGCT GCCGGAGCTCTGCAGATATCCTATCTGAATCATC GCCGGAGCTCTGCAGATATCGGTCACAAAACGGGA

**Table 5:** Sequence coverage of rotavirus genomes using viral capture and amplification with random and specific primers

Sample ID	GP type	Genome Coverage (%)	VP7	VP4	VP6	VP1	VP2	VP3	NSP1	NSP2	NSP3	NSP4	NSP5
			Segment length and coverage (%)										
VN-0172	G1P[8]	94.93	1008 (94.9)	2141 (90.6)	1356 (100)	3241 (98.2)	2690 (100)	2230 (86.1)	1566 (97.2)	1059 (100)	1074 (97.3)	570 (75.9)	640 (96.0)
VN-0341	G1P[8]	93.05	1012 (95.3)	2338 (99.0)	1356 (100)	3203 (97.0)	2644 (98.3)	2513 (97.0)	1260 (78.2)	1058 (99.9)	985 (89.2)	581 (77.4)	315 (47.2)
VN-0221	UT	92.52	995 (93.7)	2016 (85.4)	1168 (86.1)	3261 (98.8)	2564 (95.3)	2266 (87.5)	1482 (92.0)	1059 (100)	956 (86.6)	748 (99.6)	652 (97.8)
VN-0181	UT	91.07	800 (75.3)	1678 (71.0)	1356 (100)	3222 (97.6)	2593 (96.4)	2328 (89.9)	1418 (88.0)	1059 (100)	908 (82.2)	746 (99.3)	667 (100)
VN-0058	G26P[19]	92.26	1062 (100)	2337 (98.9)	1309 (96.5)	3250 (98.4)	2321 (86.3)	2183 (84.3)	1247 (77.4)	1001 (94.5)	1037 (93.9)	717 (95.5)	654 (98.1)
VN-0132	G3P[4]	92.64	1060 (99.8)	2220 (93.9)	1294 (95.4)	3090 (93.6)	2690 (100)	2086 (80.5)	1452 (90.1)	996 (94.1)	1074 (97.3)	626 (83.4)	562 (84.3)
VN-0006	G1P[8]	95.87	1060 (99.8)	2338 (99.0)	1356 (100)	3050 (92.4)	2690 (100)	2591 (100)	1424 (88.4)	1029 (97.2)	1029 (93.2)	652 (86.8)	486 (72.8)

### Novel insights into rotavirus infections and phylogenetics

Once the purification and amplification for RoV was optimised, we performed analysis on the sequences of the 26 RoV positive samples that had been generated through the modified sequencing protocol. All samples had high coverage, suitable for further downstream sequence and phylogenetic analyses. Firstly, we used the conventional loci (VP4 and VP7) to genotype the 26 samples into G and P types. We were able to determine the genotype in all 26 samples, with 22/26 corresponding with the original predicted genotype that was determined using conventional genotyping methods and 4/26 samples providing discrepant results. We found that these four samples were identified to have untypeable RoV by initial genotyping (VN-0221, VN-0186, VN-0175 and VN-0181), while genome sequencing revealed that these infections were induced by RoV genotypes G2P[4], G2P[4], G2P[4] and G1P[4], respectively.

**Fig. 3:**
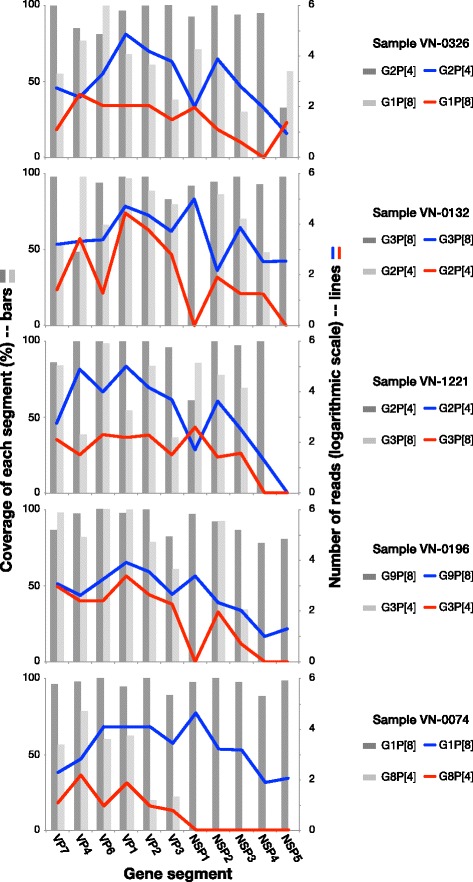
Rotavirus coinfection is characterized by a major and a minor population. Dark grey bars and blue lines indicate the percentage sequence coverage and total number of reads (log scale), respectively, for each of the 11 RoV segments for the majority population present in five samples containing coinfecting RoV populations. Light grey bars and red lines indicate the percentage sequence coverage and total number of reads (log scale), respectively, for each of the 11 rotavirus A segments for the minority rotavirus population present in five samples containing coinfecting rotavirus populations

We further found that 5/26 samples produced genome sequences that included multiple sequences of differing genotypes for one or more individual segments. Hypothesizing co-infection, we examined the varying segments and the potential genome constellations of the sequences generated via the capture-amplification method (VN-0326, VN-0132, VN-1221, VN-0196 and VN-0074). Given the segment coverage and the most likely segment combinations to form genome constellations (Fig. 2), these samples were found to be probable co-infections of G1P[8]/G2P[4], G3P[8]/G2P[4], G2P[4]/G3P[8], G9P[8]/G8P[4] and G1P[8]/G8P[4]. In all examples, we could detect a major and minor RoV genotype in the sequences (major variant appearing first in the above description (Fig. 3)), with segment coverage roughly associating with read depth. Notably, in all of these co-infection samples, we did not generate sequences for all segments, particularly the shorter segments which were again underrepresented. NSP4 was missing in the minor RoV genotype in all samples and all non-structural protein segments were missing in the minor variant in sample VN-0074.

**Fig. 4:**
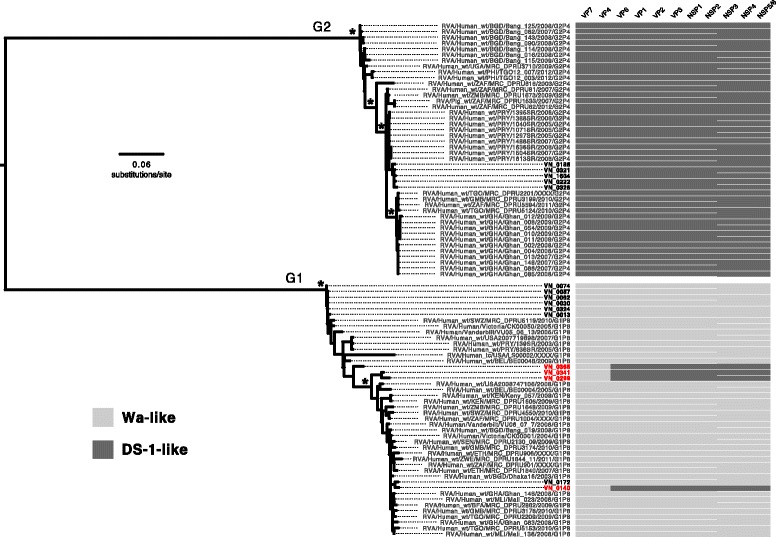
Genome constellations suggest intergenotype reassortment in Vietnamese G1P[8] and G2P[4] rotaviruses. A maximum likelihood phylogeny of VP7 gene sequences is shown with genome constellations mapped at the tips of the tree. Standard Wa-like genome segments are indicated by light grey blocks, DS-1-like segments by dark grey blocks. Taxa names in bold indicate Vietnamese sequences from this study; taxa names in red indicate likely reassortant Vietnamese sequences. Asterisks indicate ≥85% bootstrap support at internal nodes of interest

**Fig. 5:**
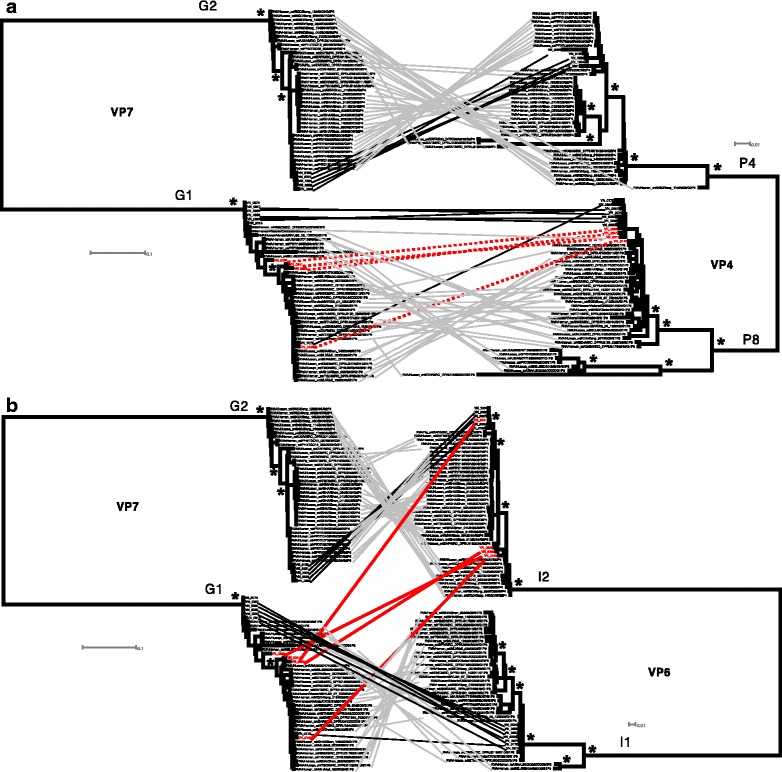
Phylogenetic incongruity among rotavirus segments confirming genomic reassortment. Diagram outlines G1P[8] VP7-VP4 segments and the DS-1-like backbone segments in four Vietnamese G1P[8] rotaviruses. **a** Tanglegram showing corresponding VP7 and VP4 segments of G1P[8] and G2P[4] genotypes. **b** Tanglegram showing phylogenetic incongruity between the VP7 and VP6 segments of the G1P[8] and G2P[4] genotype for four reassortant rotaviruses from Vietnam. Sequences from nonreassortant Vietnamese rotaviruses are indicated by connection with black lines. Sequences from reassortant Vietnamese viruses are indicated by connection with red lines. Segments between which no reassortment is detected (i.e. VP7-VP4) are depicted with reassortant viruses connected by red dashed lines. Asterisks indicate ≥85% bootstrap support at internal nodes of interest

Finally, to examine potentially reassortant G1P[8] sequences (VN-0140, VN-0299, VN-0341, VN-0365), we performed segment-specific phylogenetic analyses of G1P[8] and G2P[4] sequences with standard Wa-like or DS-1-like backbone constellations [21], including 16 samples sequenced herein. Maximum likelihood phylogenies demonstrated strong support for two different lineages (Wa-like versus DS-1-like) for each of the segments (Additional file 1: Figure S1). This divergence is highlighted in the VP7 phylogeny (Fig. 4), where the G1 and G2 genotypes form two distinct, well-supported clades. The corresponding visualization of genome constellations shows an association between the G2 genotype and the DS-1-like backbone as well as the G1 genotype and the Wa-like backbone for all but four of the Vietnamese sequences. These sequences (VN-0140, VN-0299, VN-0341, VN-0365) instead show G1P[8] associated with a DS-1-like backbone. Comparison of the phylogeny of VP7 to those of VP4 and VP6 captures the phylogenetic incongruity between segment-specific phylogenies (Fig. 5). Mixing of reassortant virus segments can be additionally observed in the VP7-VP6 tanglegram, while the VP7-VP4 tanglegram shows VP7 and VP4 sequences to be associated by genotype, suggesting a common phylogenetic history. A comparison between VP7 and all other segments is shown in Additional file 1: Figure S1. While reassortment among RoV segments is not uncommon, standard RoV genotyping only captures reassortment between the VP7 and VP4 segments, and suggests that these infections resulted from typical G1P[8] RoV.

## Discussion

Realising the need for a reliable approach for producing RoV genome sequences without the requirement for viral culture, we have developed a methodology that reproducibly generates RoV genome sequences directly from infected human fecal specimens, producing nearly complete genomes (>90% genome coverage) for 100% of the 26 samples tested. This represents a significant improvement over previous full genome sequencing approaches utilized for large-scale evolutionary analysis, which yielded nearly complete genomes in less than 45% of sequencing attempts [17, 22, 23]. Our novel approach bypasses many of the current restrictions for generating virus-specific genome sequences directly from fecal material. Further, our method reduces contamination with non-RoV sequences that may overwhelm the final sequence output, which can occur using non-selective nucleic acid purification and amplification procedures prior to sequencing. By using diagnostic ProspecT RoV EIA plates, with an adapted methodology, we show that RoV can be reliably purified in appropriate concentrations for downstream amplification and genomic analyses. These EIA plates are commercially available, and the World Health Organization (WHO) RoV Surveillance Network has included these kits in the WHO-GSM (Global Management System) catalogue for easy procurement for participating RoV surveillance network laboratories. Therefore, our technique could theoretically be rolled out throughout participating RoV Surveillance Network laboratories following routine diagnosis. We additionally suggest that the presented method is more simplistic than other genome sequencing approaches and could be performed for RoV or other viral diarrheal pathogens (kits are available for norovirus, astrovirus and other enteric pathogens), in a basic molecular virology/microbiology laboratory with access to a thermal cycling machine. Furthermore, given the high affinity for RoV particles on the EIA plates and the likely stability post-purification, we predict that RoV capture prior to extraction and amplification could be performed at field sites and shipped to a central local or international reference laboratory for direct amplification and genome sequencing.

Current RoV surveillance is largely performed using G/P typing alone, which is dependent on PCR amplification and sequencing of the genes encoding the VP4 and VP7 surface antigens, respectively. G/P typing conventionally utilises Sanger sequencing and is unable to determine a G/P type for all RoV positive fecal specimens. Indeed, in 2013, the WHO RoV Surveillance Network reported 0.6% to 9.1% prevalence of untypable RoV strains in fecal specimens depending on the location [24]. Our findings suggest that untypable RoV strains may be an artefact of methodology created via an inability to generate reliable PCR amplicons for the VP4 and VP7 genes from fecal extractions. Here we found that 4/24 previously untypable RoV strains were actually conventional G2P[4] and G1P[4] strains for which we could not produce a PCR amplicon suitable for conventional sequencing. We predict that the purification and genome sequencing of additional untypable samples would generate new insights into the global epidemiology and strain diversity of RoV. Our ability to generate genome sequences from a wide range of genotypes suggests that this methodology has a good utility and, given the global introduction of RoV vaccination, would assist in the development of a robust and expandable system of molecular epidemiology through routine RoV surveillance.

Given the global relevance of RoV and likely impact of RoV immunization in coming years, there is a paucity of genomic data for this ubiquitous RNA virus in comparison to other RNA viruses that have a dramatic impact on global health. Much of our inference of RoV strain circulation, epidemiology and evolution has been derived from current inadequate typing methods. Our data, whilst generated from a small sample size, predicts that coinfection and reassortment are common and likely go undetected at a large scale. We could identify and confirm (by specific sequences) four differing RoV combinations contributing to mixed infections (G1P[8]/G2P[4], G3P[8]/G2P[4], G9P[8]/G8P[4] and G1P[8]/G8P[4]). We additionally identified four reassortant viruses (G1P[8] with a DS-1-like backbone) in this restricted cross-section of RoV sequences. There are limited data regarding both mixed infection and reassortment, although both of these biological processes are thought to play an important role in the generation of new variants and may affect pathogenesis and disease phenotype. Current models of RoV evolution predict that while VP7 and VP4 segments reassort frequently, reassortment is less common amongst the segments comprising the genomic backbone, which likely maintain preferred genome constellations determined by protein-protein interactions across different segments [17, 22]. However, the frequency and genomic dynamics of mixed infection, a prerequisite for reassortment, are poorly understood due to a lack of adequate methods to characterize multiple co-infecting viral segments in a single sample. The method described here could be utilized to fill this knowledge gap by providing full RoV genomic data at an epidemiological scale, which would allow for a more accurate characterization of circulating RoV, investigation of mixed infection and reassortment dynamics, and analysis of RoV genomic diversity and dynamics pre- and post-vaccine introduction.

There are some limitations to our methodology that may necessitate some optimization prior to the universal acceptance of this sequencing strategy. First, the generation of double-stranded cDNA, whilst reliable, is relatively laborious and could be streamlined by using commercially available kits to provide greater utility in field locations. Second, the methodology proved to be reliable only on samples with a Ct value of <30, which may limit the study of prolonged infections and fecal carriage, which have been described to have a lower viral load than acute infections [25, 26]. Third, we validated the method on a cohort of young children with symptomatic infections in Vietnam and the method may need further testing on archived and non-archived samples from differing locations where RoV strain circulation may be more variable. Lastly, the use of Illumina sequencing makes the outlined method more costly than current genotyping methods. However, given the wealth of data generated and the decreasing costs of next generation sequencing, we present the proposed method as a first step toward a reproducible system for investigating global RoV molecular epidemiology and surveillance of RoV population structures pre- and post-vaccine introduction.

## Conclusions

We have developed and evaluated a genome sequencing method for RoV from human fecal specimens using commercial available EIA plates and a comparatively simple assortment of molecular biology reagents. The methodology is reliable but requires further validation in a broader context and may provide new biological and epidemiological insights into RoV strain diversity and disease phenotype.

## Methods

### Clinical specimens and detection of rotavirus

The fecal specimens used in this study were collected from children recruited into a randomized controlled trial for probiotics treatment conducted at Children’s Hospital Two in Ho Chi Minh City (HCMC), Vietnam. A full description of the methods has been published elsewhere [27]. Hospitalised patients between 9 and 60 months of age with acute, non-bloody, non-mucoid, watery diarrhoea and a history of less than three days were eligible to enter the trial. Patients could not enter the trial if they had at least one episode of diarrhoeal disease in the month prior to admission, they were known to have short bowel syndrome or chronic (inflammatory) gastrointestinal disease, they were immunocompromised or immunosuppressed, they were on prolonged steroid therapy or if they were diagnosed as severely dehydrated. Diarrhea was defined as three watery or loose stools within 24 h or one episode of bloody and/or mucoid diarrhoea [28].

After collection, fresh fecal samples were stored on site and transported to Oxford University Clinical Research Unit in HCMC. All fecal samples were subject to molecular testing to detect RoV and norovirus as previously described [29]. Briefly, total RNA was extracted from fecal samples, reverse transcribed into cDNA and used as template for real-time PCR. Fecal samples were stored at −80 °C. For real-time quantitative PCR, amplifications were performed using RNA Master hydrolysis probes (Roche Applied Sciences, West Sussex, United Kingdom) and optimized with 1.4 μl of activator on a LightCycler 480II (Roche Applied Sciences, Mannheim, Germany). Five μl of RNA was mixed with 20 μM for each primer and 10 μM probe, and thermal cycling was initiated at 61 °C for 5 min for reverse transcription, 5 min at 95 °C for amplification, and then by 45 cycles at 95 °C for 5 s and 60 °C for 45 s. Copy number of the target sequence was inferred using a standard curve generated as previously described [29].

### Preparation of rotavirus nucleic acid for genome sequencing

The procedure for this RoV extraction is shown in Fig. 1. RoV-positive fecal samples were diluted 1:1 with sterile DNase treated phosphate-buffered saline prior to mixing. Four hundred μl of fecal suspension was subjected to centrifugation at 10,621 × g in a benchtop microfuge for 10 min to sediment cellular debris, bacteria and mitochondria. The supernatants (containing viral particles) were removed by sterile Pasteur pipette and 20U of TURBO DNase (Ambion, Warrington, UK) was added to 200 μl of the resulting solution prior to incubation at 37 °C for 1 h to eliminate exogenous DNA. The DNase-treated supernatant was inoculated into the wells of ProspecT RoV EIA plates (Oxoid, Basingstoke, UK) using twice the volume recommended by the manufacturer along with the RoV-specific polyclonal antibody conjugated to horseradish peroxidase. RoV antigen in the sample was captured between the solid phase antibody on the plate surface and the enzyme-conjugated antibody. After one-hour incubation at ambient temperature, the wells on the EIA plates samples were washed nine times with washing buffer contained in the kit and three times with phosphate buffered saline.

After washing to remove unattached viral particles and other contaminants 750 μl of TRIzol LS (Life Technologies, Paisley, UK) was added into each of the wells on the EIA plate. The resulting lysate was removed from the EIA plates and used as the input material for a conventional TRIzol extraction following the manufacturer’s instructions. The resulting pellet of nucleic acid was resuspended in 20 μl of nuclease-free water (Ambion, Warrington, UK) prior to the removal of residual DNA with a secondary addition of TURBO DNase (Ambion, Warrington, UK) after incubation at 37 °C for 1 h. Reverse transcription with Superscript III (Invitrogen) was performed as previously described [20], using the modified primer FR26RVN (a 20-bp primer sequence with nonribosomal random hexamers at the 3′ end) at a concentration of 1 μM (Table 4) [30]. We further aimed to improve the coverage of the short RoV genome segments and reduce contaminating sequences using a combination of the random primer FR26RVN (1 μM) and FR26R-specific primers (20 nM). We additionally exploited two conserved terminal sequences for each segment [31] to generate specific primers for each of the 11 segments (Table 4).

For PCR amplification prior to sequencing, 8 μL of extracted RNA was mixed with 1 μl of a solution containing dNTPs at 10 mM, 3 μl of modified FR26RVN primer and 1 μL of mixture of FR26RV-specific primers (Table 4). The solution was incubated at 65 °C for 5 min and then placed on ice. A reaction mix of 7 μl containing 4 μl of 5x buffer (Invitrogen, Paisley, UK), 1 μl of 0.1 M DTT (Invitrogen, Paisley, UK), 1 μl of recombinant RNase inhibitor (Invitrogen, Paisley, UK) and 1 μl of reverse transcriptase Superscript III (Invitrogen, Paisley, UK) was added. The reaction was incubated at 25 °C for 10 min, 37 °C for 50 min and 75 °C for 15 min. Subsequently, second strand DNA synthesis was performed with 5 U of Klenow fragment 3′-5′ exo – (New England Biolabs, Hitchin, UK) at 37 °C for 60 min. A final incubation at 75 °C for 10 min was performed to terminate the Klenow reaction. Finally, random PCR was used to produce a sufficient yield of DNA from the ds cDNA template for Illumina sequencing. A 50 μl PCR reaction mix consisted of 45 μl Platinum PCR Supermix High Fidelity (Invitrogen, Paisley, UK), 1 μM of the primer FR20RV (GCCGGAGCTCTGCAGATATC) and 4 μl of the ds cDNA template. After 2 min at 94 °C, the reaction underwent 40 cycles of amplification (94 °C for 30 s, 60 °C for 1 min, and 72 °C for 2 min).

### Genome sequencing

The Nextera XT DNA Library Preparation kit (Illumina, Cambridge, UK) was used to generate the sequencing library. After purification using the Agencourt AMPure XP PCR kit (Beckman Coulter, Krefeld, Germany), the purified DNA library was quantified using Qubit dsDNA HS Assay kit (Life Technologies, Paisley, UK), a fluorometric-based method specific for duplex DNA quantification; the sample was then diluted to 0.2 ng/μl. Five μl of input DNA (1 ng total) was tagmented (tagged and fragmented) by the Nextera XT transposome to add unique adapter sequences. Subsequently, a limited-cycle PCR reaction targeted these adapters to amplify insert DNA and add index sequences on both ends of the DNA, thus enabling dual-indexed sequencing of pooled libraries. Samples were pooled and Kappa PCR (Kapa Biosystems, West Sussex, UK) was performed before pooling to determine the quantity of each library in the pooled sample. Sequencing was performed with 24 samples multiplexed per run on a MiSeq platform (Illumina) for 300 cycles (150-bp paired-end reads).

### Sequence analysis

Before analysing the generated RoV sequences, some simple quality control measures were always performed to ensure that raw data were of sufficient quality and there were no complications or biases in the raw sequence data. FastQC software [32] (http://www.bioinformatics.babraham.ac.uk/projects/fastqc/) was used to assess overall sequence quality. Raw Illumina paired-end reads were trimmed of adapter sequences and quality filtered using Trim Galore (http://www.bioinformatics.babraham.ac.uk/projects/trim_galore/). To identify the nearest RoV reference strain for each sample, the BLAST toolkit was used to search for the best match in the NCBI nucleotide database for every read using the MEGABLAST option [33]. The filtered sequence data were then mapped against a database of RoV reference genes using SMALT (http://www.sanger.ac.uk/science/tools/smalt-0). RoV reference files contained segment-specific sequences representing all of the known RoV diversity downloaded from GenBank using BLAST searches of the reference sequences of all known RoV genotypes. Segment-specific consensus sequences were constructed from read mapping (BAM) based on simple majority rules using script bam2cons.py written in Python from ViPR software (https://github.com/CSB5/vipr). The bam2cons_iter.sh uses BWA to do iterative mapping of the reads to the reference sequence until a consensus is generated based on the maximum frequency of nucleotide at a given position [34]. This process was conducted individually for each of the eleven RoV segments. LoFreq2 was then used to detect the single nucleotide variants present in the sample [35]. Visualization of the genome coverage graph for the segments of interest and SNPs identified by LoFreq2 were plotted using Circos software [36]. In cases of multi-genotype mixed infections, a major and minor population was called based on the number of reads for each segment and the expected genotypes given the G-P (VP7/VP4) types present in the sample, with the genome constellation with the highest number of reads referred to as the major population and that with the lowest number of reads referred to as the minor population.

### Phylogenetic analysis

Among the sequences determined here, we observed four genome sequences representing potential reassortants (samples VN-0140, VN-0299, VN-0341, VN-0365), possessing G1P8 capsid genes (VP7 and VP4) with a DS-1-like backbone (I2-R2-C2-M2-A2-N2-T2-E2-H2) (Fig. 3). To further investigate these sequences, segment-specific databases were compiled for the potential reassortants, Vietnamese G1P8 and G2P4 sequences with a standard Wa-like or DS-1-like genome constellation, and a representative subsample of G1P8 RoV with a Wa-like backbone (I1-R1-C1-M1-A1-N1-T1-E1-H1) and G2P4 RoV with a DS-1-like backbone were obtained from the Virus Pathogen Resource (ViPR) database (http://www.viprbrc.org/), and manually aligned using Geneious (v9.0). Segment-specific maximum likelihood (ML) trees were then inferred using RAxML [37] under the GTRGAMMA model with 500 bootstrap replications; this model was chosen for computational simplicity, as the best-fit nucleotide substitution model for each dataset could be approximated by or was nested in the GTRGAMMA model, as determined using jModelTest [38]. These trees were then utilized to generate tanglegrams for visualizing the relationships between reassortant segments using Dendroscope 3 [39].

## Additional file

Additional file 1: Figure S1. Phylogenetic incongruity confirms reassortment of G1P[8] VP7-VP4 segments and DS-1-like backbone segments in four Vietnamese G1P[8] rotaviruses. (A) Tanglegram showing correspondence across VP7 and VP4 segments of the G1P[8] and G2P[4] genotype. (B) Tanglegram showing phylogenetic incongruity for the VP7 and VP6 segments of the G1P[8] and G2P[4] genotype for four reassortant rotaviruses from Vietnam. (C) Phylogenetic incongruity in VP7 and VP1. (D) Phylogenetic incongruity in VP7 and VP2. (E) Phylogenetic incongruity in VP7 and VP3. (F) Phylogenetic incongruity in VP7 and NSP1. (G) Phylogenetic incongruity in VP7 and NSP2. (H) Phylogenetic incongruity in VP7 and NSP3. (I) Phylogenetic incongruity in VP7 and NSP4. (J) Phylogenetic incongruity in VP7 and NSP5. In all tanglegrams, sequences from nonreassortant Vietnamese rotaviruses are indicated by connection with black lines. Sequences from reassortant Vietnamese viruses are indicated by connection with red lines. Segments between which no reassortment is detected (i.e. VP7-VP4) are depicted with reassortant viruses connected by red dashed lines. Asterisks indicate ≥85% bootstrap support at internal nodes of interest. (PDF 3252 kb)

## Abbreviations

DALYs: Disability adjusted life years
dsRNA: Double-stranded RNA
EIA: Enzyme immunoassay
ENA: European Nucleotide Archive
GSM: Global Management System
HCMC: Ho Chi Minh City
IQR: Interquartile range
NGS: Next generation sequencing
PCR: Polymerase chain reaction
RT-PCR: Reverse transcriptase polymerase chain reaction
WHO: World Health Organization
