# Supplementary figures and images for: A universal genome sequencing method for rotavirus A from human fecal samples which identifies segment reassortment and multi-genotype mixed infection
Source: BMC Genomics. 2017 Apr 24;18:324. doi: 10.1186/s12864-017-3714-6 (PMC5404283; doi:10.1186/s12864-017-3714-6)

(A)

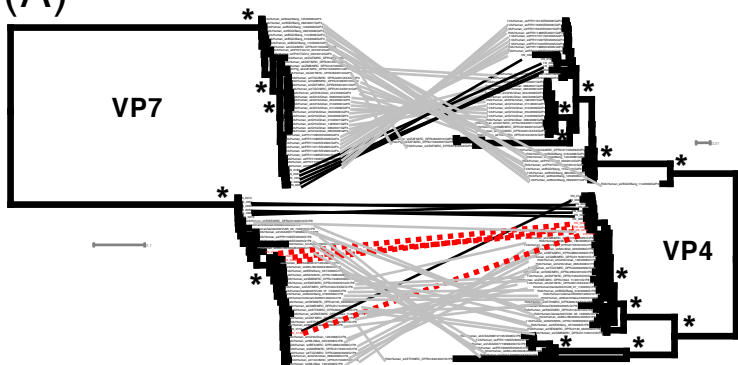

(B)

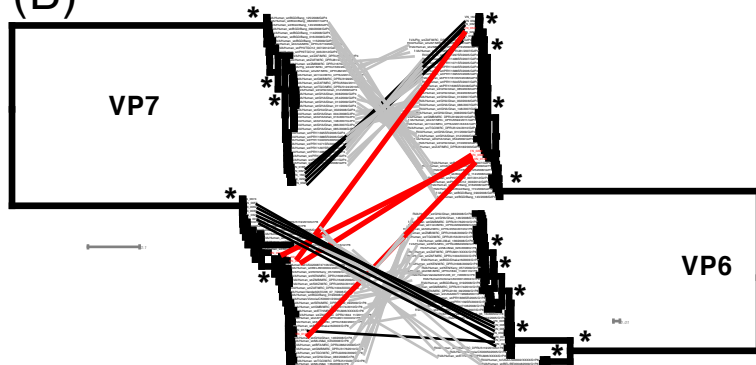

(C)

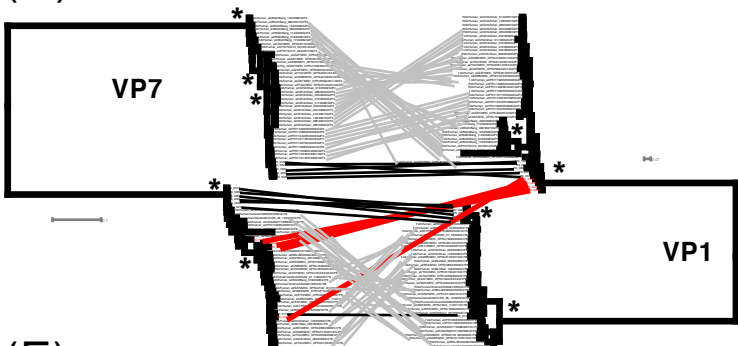

(D)

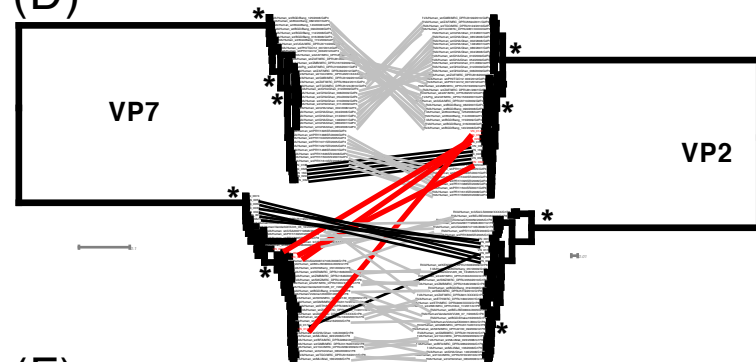

(E)

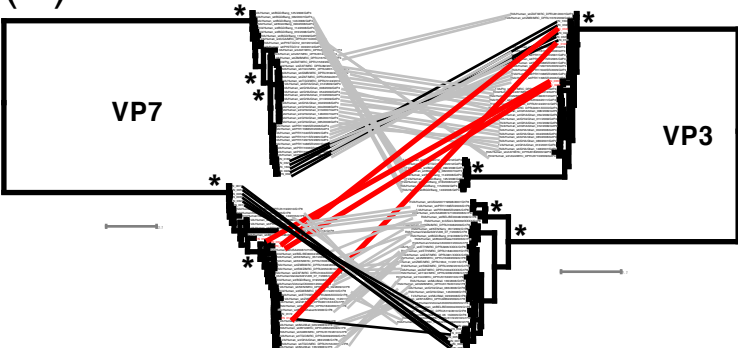

(F)

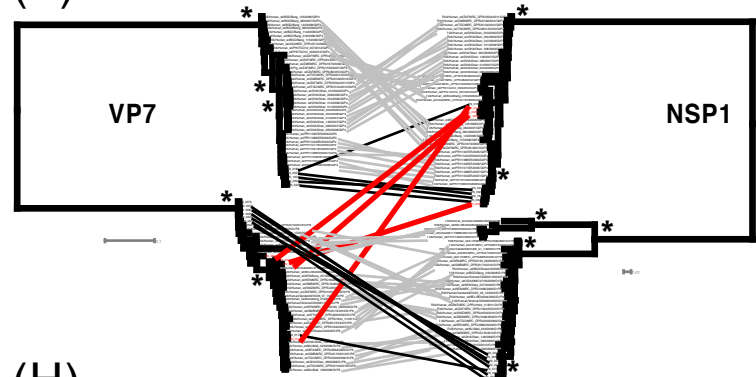

(G)

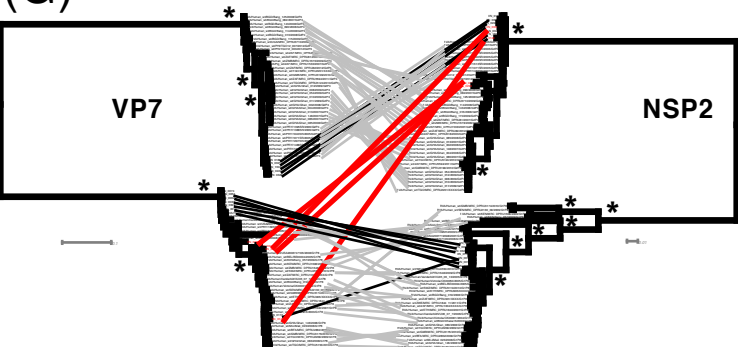

(H)

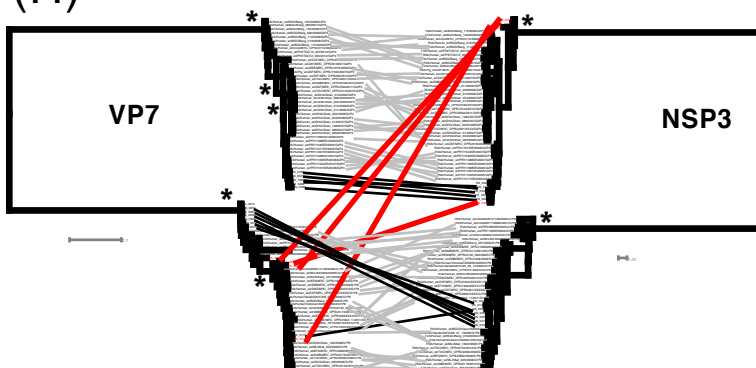

(I)

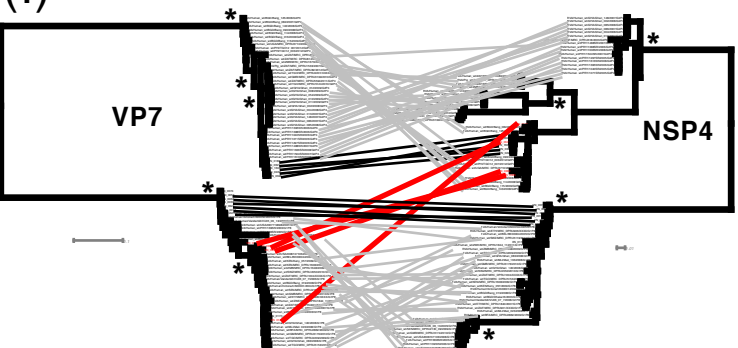

(J)

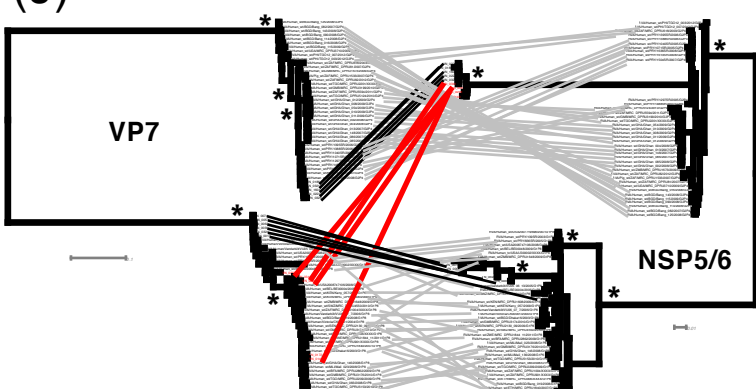

Supplement: Additional file 1: Figure S1. — Phylogenetic incongruity confirms reassortment of G1P[8] VP7-VP4 segments and DS-1-like backbone segments in four Vietnamese G1P[8] rotaviruses. (A) Tanglegram showing correspondence across VP7 and VP4 segments of the G1P[8] and G2P[4] genotype. (B) Tanglegram showing phylogenetic incongruity for the VP7 and VP6 segments of the G1P[8] and G2P[4] genotype for four reassortant rotaviruses from Vietnam. (C) Phylogenetic incongruity in VP7 and VP1. (D) Phylogenetic incongruity in VP7 and VP2. (E) Phylogenetic incongruity in VP7 and VP3. (F) Phylogenetic incongruity in VP7 and NSP1. (G) Phylogenetic incongruity in VP7 and NSP2. (H) Phylogenetic incongruity in VP7 and NSP3. (I) Phylogenetic incongruity in VP7 and NSP4. (J) Phylogenetic incongruity in VP7 and NSP5. In all tanglegrams, sequences from nonreassortant Vietnamese rotaviruses are indicated by connection with black lines. Sequences from reassortant Vietnamese viruses are indicated by connection with red lines. Segments between which no reassortment is detected (i.e. VP7-VP4) are depicted with reassortant viruses connected by red dashed lines. Asterisks indicate ≥85% bootstrap support at internal nodes of interest. (PDF 3252 kb) [file 12864_2017_3714_MOESM1_ESM.pdf]
